# The complete chloroplast genome of *Lemmaphyllum intermedium*, a valuable medicinal fern

**DOI:** 10.1080/23802359.2020.1870888

**Published:** 2021-02-09

**Authors:** Zhen Wang, Ning Li, Zhan Li, Yujia Liu, Ping Yang, Yongfeng Hong, Ziqing He, Yingjuan Su, Ting Wang

**Affiliations:** a School of Life Sciences, Sun Yat-sen University, Guangzhou, China; b Research Institute of Sun Yat-sen University in Shenzhen, Shenzhen, China; c College of Life Sciences, South China Agricultural University, Guangzhou, China

**Keywords:** *Lemmaphyllum intermedium*, chloroplast genome, phylogenetic analysis

## Abstract

*Lemmaphyllum intermedium* is a valuable medicinal fern in China. We have determined its complete chloroplast genome using Illumina paired-end sequencing. The genome is 157,795 bp in length with 132 genes, including 88 protein-coding genes, eight ribosomal RNA genes, 35 tRNA genes, and one pseudogene. It has a quadripartite structure consisting of a small single-copy (SSC) of 21,764 bp, a large single-copy (LSC) of 81,189 bp, and two inverted repeats (IRs) of 27,421 bp each. A maximum-likelihood phylogenetic tree reconstructed by using complete chloroplast genome sequences reveals that *L. intermedium* is closely related to *Lemmaphyllum carnosum* var. *microphyllum* with strong support.

*Lemmaphyllum intermedium* (Ching) Li Wang, also known as *Lemmaphyllum diversum* (Rosenstock) Tagawa, is a vigorous fern belonging to Polypodiaceae (Zhang et al. 2013). The plant grows on rocks under the forest canopy at the altitude of 700 to 2200 m. Its rhizomes are covered with brown and subulate-lanceolate scales, and sporangia in discrete sori is arranged in a line on each side of midrib (Zhang et al. 2013). In China, *L. intermedium* is mainly distributed in provinces Shanxi, Gansu, Hubei, Sichuan, and Guizhou (Zhang et al. 2013). The whole plant of the fern is used in traditional Chinese medicine for inflammation, arthralgia, and bleeding stopping (Zhang et al. 2013). There exists taxonomic ambiguity for genera *Lemmaphyllum*, *Weatherbya, Lepisorus*, and *Caobangia* due to morphological similarity (Wang et al. 2010; Wei et al. 2017). The phylogenetic position of *Lemmaphyllum* and *Lepidogrammitis* remains in disputation (Wang et al. 2010). More importantly, the species delimitation in *Lemmaphyllum* is still an unresolved issue (Wei and Zhang 2013). Therefore, characterization of the whole chloroplast genome sequence of *L. intermedium* will be helpful to clarify the phylogeny and taxonomy of *Lemmaphyllum*.

Total genomic DNA was isolated from fresh and healthy fronds of *L. intermedium* collected from South China Botanical Garden, Chinese Academy of Sciences (23°11′3.56″N, 113°21′43.28″E). The specimen was deposited in the Herbarium of Sun Yat-sen University (SYS; voucher: SS Liu *20161024*). We constructed a shotgun library with approximate insert lengths of 300 bp and performed a high-throughput sequencing on Hiseq 2500 platform (Illumina Inc., San Diego, CA). A total of 2.66 G pair-end raw data were generated. Trimmomatic v0.32 (Bolger et al. 2014) and FastQC v0.10.0 (Andrews 2010) were used for quality-trimming and assess. Then the obtained clean data were *de novo* assembled with Velvet v1.2.07 (Zerbino and Birney 2008). The assembled chloroplast genome was annotated by using GeSeq (Tillich et al. 2017) and tRNAscan-SE programs (Schattner et al. 2005), and corrected by blasting search against *Lepisorus clathratus* and *Lemmaphyllum carnosum* var. *microphyllum* (GenBank: KY419704 and MN623356) as references. To conduct phylogenetic analyses the complete chloroplast genome sequences of 12 ferns were retrieved from the National Center for Biotechnology Information (NCBI), and the sequence alignment was created by using the MAFFT v7.311 (Katoh and Standley 2013). A maximum likelihood (ML) tree was constructed with RAxML v8.2.12 (Stamatakis 2014) using *Alsophila spinulosa* as outgroup. Bootstrap values were computed from 1000 replicates.

The chloroplast genome of *L. intermedium* is 157,795 bp in length with 132 genes (accession number: MT968973), including 88 protein-coding genes, eight ribosomal RNA genes, 35 tRNA genes, and one pseudogene. It has a typical quadripartite structure composed by a small single-copy (SSC, 21,764 bp), a large single-copy (LSC, 81,189 bp), and two inverted repeats (IRa and IRb, 27,421 bp each). Its GC content of whole genome, LSC, SSC, and IR is 42%, 41%, 38%, and 45%, respectively. There are one or two introns occurring in 18 genes: *ndh*B, *rps*16, *atp*F, *rpo*C1, *ycf*3 (2), *clp*P (2), *pet*B, *pet*D, *rps*12 (2), *ndh*A, *rpl*16, *rpl*2, *trn*G-UCC, *trn*V-UAC, *trn*A-UGC, *trn*I-GAU, *trn*L-UAA, and *trn*T-UGU. Fourteen genes are duplicated in the IR regions, which are *rps*12, *rps*7, *psb*A, *ycf*2, *trn*N-GUU, *trn*H-GUG, *trn*I-GAU, *trn*A-UGC, *trn*T-UGU, *trn*R-ACG, *rrn*4.5, *rrn*5, *rrn*16, and *rrn*23. ML tree reveals that *L. intermedium* is sister to *L. carnosum* var. *microphyllum* with a high bootstrap value (Figure 1). Our chloroplast genome sequencing provides essential data for the phylogenetic characterization of *Lemmaphyllum* and its related genera.

**Figure 1.**
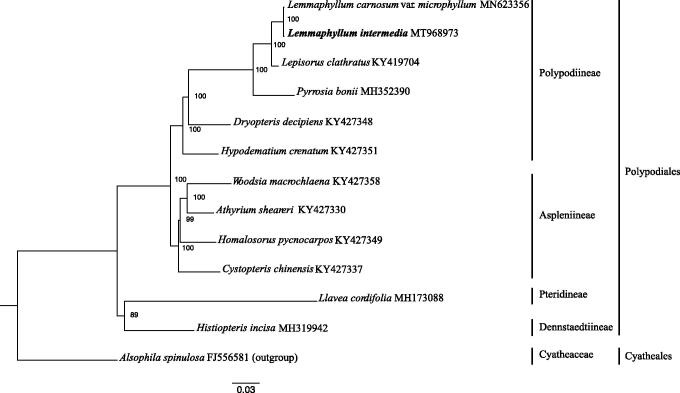
The phylogenetic tree including *Lemmaphyllum intermedium* reconstructed based on whole chloroplast genome sequences by using maximum likelihood method with *Alsophila spinulosa* as the outgroup. Bootstrap scores are indicated for each branch.

## Data Availability

The data that support the findings of this study are openly available in GenBank of NCBI at https://www.ncbi.nlm.nih.gov/nuccore/MT968973, GenBank accession number MT968973. Raw sequencing reads in this study are deposited in https://www.ncbi.nlm.nih.gov/sra/SRR12989517, with SRA number SRR12989517.
